# Predicting the behavioral impact of transcranial direct current stimulation: issues and limitations

**DOI:** 10.3389/fnhum.2013.00613

**Published:** 2013-10-04

**Authors:** Archy O. de Berker, Marom Bikson, Sven Bestmann

**Affiliations:** ^1^Sobell Department of Motor Neuroscience and Movement Disorders, UCL Institute of Neurology, University College LondonLondon, UK; ^2^Neural Engineering Laboratory, Department of Biomedical Engineering, The City College of New York of City University of New YorkNew York, NY, USA

**Keywords:** neuromodulation, modeling, computational neurostimulation, validation, neuroenhancement

## Abstract

The transcranial application of weak currents to the human brain has enjoyed a decade of widespread use, providing a simple and powerful tool for non-invasively altering human brain function. However, our understanding of current delivery and its impact upon neural circuitry leaves much to be desired. We argue that the credibility of conclusions drawn with transcranial direct current stimulation (tDCS) is contingent upon realistic explanations of how tDCS works, and that our present understanding of tDCS limits the technique’s use to localize function in the human brain. We outline two central issues where progress is required: the localization of currents, and predicting their functional consequence. We encourage experimenters to eschew simplistic explanations of mechanisms of transcranial current stimulation. We suggest the use of individualized current modeling, together with computational neurostimulation to inform mechanistic frameworks in which to interpret the physiological impact of tDCS. We hope that through mechanistically richer descriptions of current flow and action, insight into the biological processes by which transcranial currents influence behavior can be gained, leading to more effective stimulation protocols and empowering conclusions drawn with tDCS.

Since the demonstration that transcranial direct current stimulation (tDCS) can modulate the size of motor evoked potentials (MEPs) elicited over human primary motor cortex (Nitsche and Paulus, 2000), tDCS has become a popular approach for non-invasive neurostimulation of the human brain. The success of the technique, as gauged by the breadth of publications, is striking, being employed over numerous brain areas, in variety of behavioral tasks, and in the treatment of a wide range of diseases (Brasil-Neto, 2012; Brunoni et al., 2012). It appears, however, that the growth in reported applications for tDCS has outstripped the growth in our understanding of the mechanistic underpinnings of direct current (DC) stimulation, both in terms of current delivery and the subsequent effect of electrical fields upon the cortex.

This imbalance has been exacerbated by the impressive and *a priori *surprising trend for tDCS, particularly when the anode is placed over the cortical region of interest, to facilitate behavioral performance (but see Iuculano and Kadosh, 2013). We believe that a deeper and more critical querying of (a) where currents actually flow when one applies transcranial DC stimulation and (b) the effects upon cellular and network activity, will help focus efforts to capitalize upon the remarkable achievements of the field to date. We here briefly summarize recent work on these issues, and highlight some of the key conclusions and remaining questions that are pertinent to current and future users of tDCS.

## WHICH NEURAL STRUCTURES ARE TARGETED BY DC STIMULATION?

**FIGURE 1:**
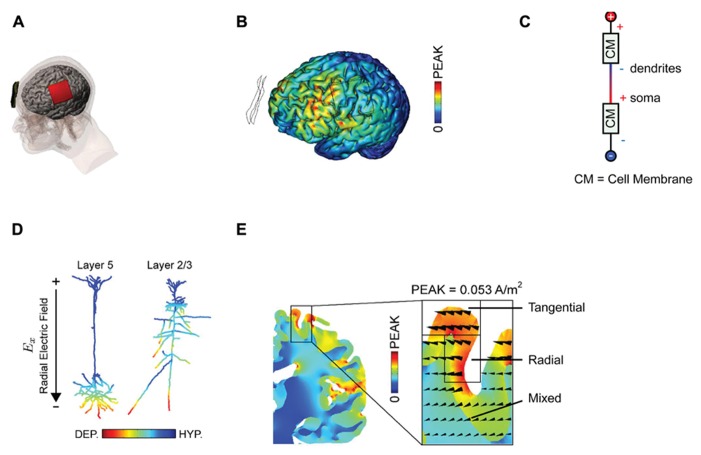
**Examining current distributions in the cortex. (A)** The classic montage used by Nitsche and Paulus (2000) to modulate MEP size. **(B)** Finite element modeling of current distribution with the classic montage illustrates the broad swathes of cortex affected. **(C)** Voltage drops across membranes considered as resistors explain the mixture of hyperpolarization and depolarization seen in. **(D)** Superficially depolarizing currents produce hyperpolarization in dendrites and depolarization in the cell soma. **(E)** The direction of current flow varies considerably within the stimulated area. All figures bar **(C)** reprinted with permission from Rahman et al. (2013).

The arrangement of anode and cathode used by Nitsche and Paulus (2000) that led to a potentiation and suppression of MEP’s is pictured in **Figure 1A**. This “classic montage” consists of a pair of sponges, one of which is placed over the motor cortex contralateral to the limb in which MEPs are measured, with the other positioned over the forehead of the opposite hemisphere. It is appropriate to recall here that in any montage using DC, an anode and a cathode will be present; which electrode is deemed the “reference” is merely a matter of convention and depends only upon the predicted effect of stimulation upon the measured behavior. It is also relevant to point out that in their original paper and subsequent work, Nitsche and co-workers do not attribute the observed change in MEP size to the stimulation of any one cortical area *per se. *Moreover, this group is careful to distinguish between montage-specific effects (e.g., the passage of current in various patterns across the brain) and focality (limited current flow to one region) (Nitsche et al., 2007). This discretion is not consistently observed in subsequent studies but is vindicated by neuroimaging data that implies that the classic montage influences numerous cortical areas (Lang et al., 2005; Zheng et al., 2011), and that standard DC stimulation protocols influence the excitability of numerous muscles (Roche et al., 2009).

One can be fairly confident, therefore, that conventional tDCS stimulation protocols, with two large electrodes placed across the head, induce widespread currents of varying intensity and therefore widespread changes in cortical activity, a conclusion further supported by numerous modeling approaches (Datta et al., 2009; Bikson et al., 2012; Ruffini et al., 2012) There is an important distinction between putting one electrode “over” a brain region, and targeting that brain region with focal current delivery – and indeed the peak brain currents in a bipolar array may be at some point between the two electrodes. This interim conclusion suggests that we are rarely in a position to make conclusive inferences about the function of a specific stimulated area based upon an observed behavioral effect alone; the impact upon networks of activity is simply too broad for topologically specific statements, though it may be reasonable to suggest behavioral effects are consistent with modulation of at least one region of interest. We note that there is a subtle distinction between this phenomenon and network effects elicited by transcranial magnetic stimulation (TMS), where the initial intervention is relatively focal, but then is likely to propagate poly-synaptically to interconnected regions (Bestmann and Feredoes, 2013). By contrast, with conventional tDCS montages the primary effects are likely to occur in a non-spatially specific way (Nitsche, 2011).

The use of mathematical, finite element models for predicting current flow offers a principled way to estimate current distributions, optimize current delivery to target areas, and to circumscribe conclusions about the brain basis of observed effects (**Figure 1B**; Dmochowski et al., 2011; Bikson et al., 2012; Miranda et al., 2013). The use of individual head models is becoming increasingly feasible with improvements in the automatization of the process, reducing the time and expertise necessary and rendering such modeling accessible to more users of tDCS (Huang et al., 2012; Windhoff et al., 2013). Although direct physiological validation of such models is limited (Datta et al., 2013; Edwards et al., 2013), at the very least this approach highlights several important issues for the delivery of transcranial current. Furthermore, the key conclusions we discuss below will not change – they are dictated by the laws of physics – though specific details of these models are likely to yield subtly different results.

Firstly, the amount and distribution of current reaching different brain regions varies wildly as a function of individual physiology and anatomy (Datta et al., 2012) contributing substantial variance to our datasets. A recent study (Edwards et al., 2013), for example, showed that models of current flow based upon individual magnetic resonance imaging (MRI) scans accurately predicted the amount of current necessary to evoke a muscle twitch with strong transcranial electrical stimulation, and successfully explained the twofold variation in evoked response when using fixed stimulus parameters. Factors such as skull thickness, distribution of cerebrospinal fluid, and subcutaneous fat (Truong et al., 2013) have a radical effect upon current flow, and add to the uncertainty about the destination and strength of transcranially delivered DC current.

Secondly, experimenters looking to increase excitability will typically place the anode over the region of interest, analogous to the positioning of the anode over M1 in the classic MEP-modulating montage. One might suppose that this equates to delivering uniform inward positive current to the cortex beneath the electrode. However, this is emphatically not the case. The topography of the cortical surface places a large role in determining current flow, with dramatic reversals in polarity between adjacent gyri and sulci (Rahman et al., 2013). It is thus simplistic to describe the entire area under the electrode as being “depolarized,” or “excited” by the application of anodal currents. Furthermore, slice studies have recently demonstrated that the cellular effect of a depolarizing current applied to the cortical surface depends upon the depth of the observed compartment (Chan et al., 1988; Bikson et al., 2004). Simply by considering the cell membrane as a resistor in a classic cable model (**Figure 1C**), it follows that when superficial compartments are hyperpolarized then deep compartments in the same cell will be depolarized – and vice versa. Each neuron will inevitably have *both *depolarized and hyperpolarized compartments during tDCS. In a pyramidal cortical neuron aligned with an inward (relative to the cortical surface) directed electric field, this equates to hyperpolarization of the dendritic tree and depolarization of the soma (**Figure 1D**; Rahman et al., 2013). Expected variations in both electric field direction and relative cell morphologies will produce variations in polarization profiles (Radman et al., 2009). Apart from revealing that any simplistic distinction between “anodal” and “cathodal” stimulation provides incomplete mechanistic grounding of behavioral and clinical DC applications, this raises another question; how does direction of current flow affect cellular responses?

Current flow is vectorial, in the sense that we can estimate both the current intensity and its direction at a given point. It is widely acknowledged that increasing total applied current density at the electrodes results in an increase in the intensity of the electrical field generated in the brain and that this affects the physiological response to tDCS (Nitsche et al., 2007). Conversely, far less discussion has focused upon the importance of current orientation (Dmochowski et al., 2012), which in each brain region is typically defined as radial (into the cortex) or tangential (parallel to the cortex). The induction of an electric field in the longitudinal axis is not electrophysiologically equivalent to one that traverses the cell (Radman et al., 2009; Rahman et al., 2013). Whilst a radially aligned inward current will cause somatic depolarization, transverse currents have pathway specific effects upon cellular activity potentially linked to polarization of afferent axons (Rahman et al., 2013). Surprisingly, given the prevalent view that we are injecting current “into” the cortex, tDCS currents are primarily tangential; they flow parallel to the cortex (Rahman et al., 2013). It is interesting to note that the cellular populations that are aligned parallel to the cortical surface, such as intracortical and interhemispheric connections, will therefore be more optimally excited with standard tDCS montages than the radially aligned cortical columns within the stimulated area.

Additionally complicating is the fact that the direction of current flow will be dramatically influenced by the pattern of sulci and gyri in the stimulated area; an electrode configuration that induces primarily radial currents in a gyrus might induce exclusively tangential ones in an adjacent sulcus (**Figure 1E**; Miranda et al., 2013; Rahman et al., 2013). Idiosyncratic different in cortex anatomy across subjects may thus produce distinct patterns of current flow and hence neuromodulation.

Taken together, this suggests that the effect of a polarizing current will differ substantially between sulci and gyri, at different depths of cortex, and between differently aligned cellular populations. It is not yet clear what the significance of this variability is in terms of the design of tDCS montages; we are not aware of any systematic exploration of this issue in humans. It might be instructive, for instance, to examine the effect of current direction upon MEP modulation, a possibility afforded by inverse-modeling that allows us to optimize montages based upon current delivery in a particular direction (Bikson et al., 2012).

## EXPLAINING THE EFFECTS OF tDCS UPON BEHAVIOR

The preceding section highlights the difficulties in delivering transcranial currents in a precise and principled manner, and the dangers of assuming homogeneity in induced currents and the simplistic predictions such assumptions spawn. However, this raises an even more fundamental question: even if we were able to deliver currents exactly as desired, it is not clear *a priori* how these would lead to the diverse and impressive behavioral effects reported in the literature. Similarly, given the complexity of current flow and limitations of achievable patterns, what is a “best” achievable current flow pattern for any given objective?

Consider the analogy of the brain as a computer, one that falls short in terms of describing the complexity and plasticity of neural networks. If one strapped a 9V battery to the processor of a laptop and improved the computer’s processing, the result would be somewhat surprising. There is no reason to believe that computers, or brains, lack electricity, which should make it very startling when injecting current improves function. It is worth, therefore, reassessing the basic hypotheses about neural processing that underlie current explanations of tDCS’s action.

Much of the current wisdom on the action of tDCS can be traced back to generalizations from the effects of DC on motor cortex. Given that we know that microstimulation of the primate motor cortex can produce MEPs (Fritsch and Hitzig, 1870), that magnetic stimulation of neurons in this area can also evoke MEPs (Hess et al., 1987), and that subthreshold stimulation of neurons in motor cortices can modulate the threshold for eliciting MEPs (Kujirai et al., 1993) it is reasonable to interpret the effects of tDCS in this area as alterations of membrane potential. Lasting effects of stimulation are consistent with the induction of long-term potentiation (LTP) between stimulated neurons, an effect that has been documented in animal models of tDCS (Márquez-Ruiz et al., 2012; Ranieri et al., 2012). It is thus parsimonious to suppose that an anodal current over M1 leads to increases in excitability and subsequently enhanced plasticity, whereas cathodal current over M1 decreases excitability and subsequent synaptic depression. This effect is plausibly attributable to depolarization of somatic membrane potential by anodal currents and hyperpolarization of soma by cathodal currents, as observed in slice studies (Chan et al., 1988; Bikson et al., 2004). A simple relationship between cellular activity and the magnitude of the evoked response renders such an explanation coherent.

It should be stressed, however, that this is an *inference*, based upon what is already known about the process of MEP production [and noting that there remains uncertainty about the neural sources controlling even this modest behavior (Di Lazzaro et al., 2008)]. Whether this can readily be generalized to other processes and other cortical areas remains unknown.

The critical point is that there is no theoretical or mechanistic explanation for why depolarizing cells would improve complex behaviors such as perceptual decision-making, mathematical ability, or motor learning. The injection of current into these processes constitutes the addition of random electrical activity that indiscriminately targets large swathes of neurons and has nothing to do with the ongoing activity pattern underlying the performance of the task at hand.

Furthermore, since subthreshold depolarization of a cell is likely to provoke plasticity of active synapses onto that cell, DC is likely to facilitate non-Hebbian plasticity (Hebb, 1949). If we accept that LTP constitutes a means for memory storage (Lisman et al., 2003), then if DC stimulation indeed produces depolarization, it will lead to the formation of irrelevant, interfering, memories. We believe that there are extensive and finely tuned mechanisms for controlling the induction and consolidation of cellular plasticity (Redondo and Morris, 2011). At risk of laboring the point, there is no *a priori* reason known to us to believe that merely depolarizing cells should make these processes more efficient, suggesting that we should challenge the simple assumption that this is what anodal tDCS does.

## COMPUTATIONAL NEUROSTIMULATION

Where does this leave us? Our understanding of tDCS at the cellular level is growing, but there remains an explanatory gap between the abstract effects of stimulation upon cells in animals/slices and the large and impressive behavioral effects documented in humans. We believe that for meaningful progress to be made we must attempt description at the appropriate level, that of neural circuits.

We provide a few selected examples that highlight the potential for mechanistically cogent accounts of tDCS function: first, the formulation of physiological hypotheses concerning plausible mechanisms whereby tDCS *might* influence cortical function to enhance processing, and, second, the use of computational modeling to describe the network consequences of stimulation (“*computational neurostimulation*”).

Stochastic resonance describes a phenomenon whereby the introduction of small amounts of noise into a non-linear system produces increases in performance when dealing with small amounts of signal (McDonnell and Ward, 2011). Schwarzkopf et al. (2011) demonstrated that the administration of low-intensity TMS to V5/MT improved discrimination in a dot-motion paradigm, an effect that was reversed at high intensities of TMS. This was interpreted as evidence that stochastic resonance plays a role in the facilitatory effects of TMS. Stochastic resonance would provide an equally compelling explanation of tDCS action; the injection of weak currents essentially constitutes the addition of neural noise. *A priori*, stochastic resonance seems a more probable outcome of tDCS than of TMS; the concerted, modulatory nature of transcranial currents make them more likely to modify existing processes than the large, abrupt disruption of normal function produced by TMS. Interestingly, a stochastic resonance account would also suggest that overstimulation might lead to a decrement in performance. Central to this suggestion is that there is a wealth of data about the underpinnings of stochastic resonance at a cellular and population level (McDonnell and Ward, 2011). Combined with individual variability in the efficacy of stimulation (Edwards et al., 2013), this might explain some of the inconsistency observed in responses to stimulation; the addition of too little or too much noise as a result of variable current delivery could produce negligible or detrimental effects upon behavior. We believe that such theoretically plausible accounts, grounded in knowledge of information processing in neural systems, may offer a productive way to enhance our understanding of tDCS action and how it should be applied in different behavioral settings.

Such efforts might be complemented by attempting to formalize the impact of stimulation upon brain networks via modeling of neural activity. Since it is *a priori* not clear why the concerted hypo- or hyperpolarization of thousands of neurons should improve the processing capacity of such populations, these models allow for exploring this issue in detail. We briefly mention two of many approaches. An elegant example for pulsed (TMS) stimulation is provided by Esser et al. (2005), who use a detailed model of thousands of neurons within a cortico-thalamic network including motor cortex, to investigate the precise impact of TMS on cortical circuits. By introducing perturbations analogous to those evoked by TMS, they were able to reproduce key features of MEPs generated by TMS administration. This approach relies on information about the effect of stimulation upon the cellular population modeled, which can then be simulated and outputs compared with empirical data.

The opposite approach, inferring the cellular perturbation from the network response, is made possible by biophysically informed network models such as dynamic causal modeling (DCM; Friston et al., 2003). A striking example of how DCMs can be used to infer the underlying changes in cellular physiology comes from Moran et al. (2011). These authors administered L-DOPA to subjects performing a working memory task, and recorded magnetoencephalography (MEG) responses. DCMs of the MEG response to drug administration recapitulated the changes in NMDA and AMPA receptor conductances known to underlie the action of dopamine in the frontal cortex (Goldman-Rakic, 1996). There is no reason why a similar approach should not be taken with tDCS, offering a rich toolkit with which to examine the neurophysiological changes underlying the immediate and delayed effects of tDCS upon behavior. Building upon combined neurostimulation and neuroimaging approaches that allow for identifying interactions between stimulation-induced behavioral change and neural activity (Siebner et al., 2009; Bestmann and Feredoes, 2013), this would involve the application of tDCS during recording of brain activity with magneto- and electroencephalography (M/EEG) or functional MRI (fMRI), and modeling of the resultant perturbation to infer the neurophysiological changes underlying the observed changes in gross brain activity and behavior.

## SUMMARY

We believe that it is imperative that our understanding of the delivery and functional impact of transcranial current continues to grow. Improving current delivery by taking into account individual anatomical variation and the complex dynamics of polarity and orientation is likely to help optimizing established therapeutic protocols. Similarly, improving and demonstrating the focality of tDCS through the use of high definition electrode arrays (HD-tDCS) (Kuo et al., 2013) might potentially make tDCS a far more valuable tool for systems neuroscientists looking to elucidate the function of specific structures. At present, experimenters should be circumspect in making claims that changes in behavior during or after tDCS are caused by an excitability change in the cortical area underlying one of the electrodes. The credibility of conclusions drawn with tDCS is contingent upon realistic explanations of how tDCS works. To this end, we promote the use of computational neurostimulation to refresh the theoretical frameworks in which to explain the impact of tDCS, in the hope of providing tDCS with a scientific credence which will also assist its use as an exploratory and therapeutic tool.

## Conflict of Interest Statement

The authors declare that the research was conducted in the absence of any commercial or financial relationships that could be construed as a potential conflict of interest.
